# UBR5 interacts with the replication fork and protects DNA replication from DNA polymerase η toxicity

**DOI:** 10.1093/nar/gkz824

**Published:** 2019-10-05

**Authors:** Lina Cipolla, Federica Bertoletti, Antonio Maffia, Chih-Chao Liang, Alan R Lehmann, Martin A Cohn, Simone Sabbioneda

**Affiliations:** 1 Istituto di Genetica Molecolare “Luigi Luca Cavalli-Sforza”, CNR, Pavia, Italy; 2 Department of Biochemistry, University of Oxford, Oxford, UK; 3 Genome Damage and Stability Centre, University of Sussex, Brighton, UK

## Abstract

Accurate DNA replication is critical for the maintenance of genome integrity and cellular survival. Cancer-associated alterations often involve key players of DNA replication and of the DNA damage-signalling cascade. Post-translational modifications play a fundamental role in coordinating replication and repair and central among them is ubiquitylation. We show that the E3 ligase UBR5 interacts with components of the replication fork, including the translesion synthesis (TLS) polymerase polη. Depletion of UBR5 leads to replication problems, such as slower S-phase progression, resulting in the accumulation of single stranded DNA. The effect of UBR5 knockdown is related to a mis-regulation in the pathway that controls the ubiquitylation of histone H2A (UbiH2A) and blocking this modification is sufficient to rescue the cells from replication problems. We show that the presence of polη is the main cause of replication defects and cell death when UBR5 is silenced. Finally, we unveil a novel interaction between polη and H2A suggesting that UbiH2A could be involved in polη recruitment to the chromatin and the regulation of TLS.

## INTRODUCTION

DNA damage poses a great threat to DNA replication. If left unrepaired it can lead to mutations, chromosomal aberrations and possibly cell death (1). For this reason, DNA repair and tolerance systems have evolved to address these issues and allow safe completion of the duplication of the genome (2,3). DNA replication requires the coordination of a large repertoire of protein complexes that have to coordinate its initiation, elongation and ultimately its conclusion (4). Among the DNA tolerance systems, DNA translesion synthesis (TLS) helps completion of DNA replication in the presence of damage by using a series of specialized DNA polymerases that can accommodate specifically template DNA distorted by altered bases (5). TLS is kept under tight control by various post-translational modifications of the Proliferating Cell Nuclear Antigen (PCNA), the DNA replication processivity factor, which also serves as a loading platform for a number of DNA repair proteins (6). When the replication fork is blocked at a damaged DNA template, single-stranded DNA (ssDNA) is exposed ahead the fork as result of the uncoupling of the blocked replication fork and the ongoing replicative DNA helicase (7). The complex of E2 ubiquitin conjugating enzyme Rad6 and E3 ubiquitin ligase Rad18 is recruited to ssDNA coated by the Replication Protein A (RPA) and mono-ubiquitylates PCNA on Lysine 164 (8–10). Mono-ubiquitylated PCNA (UbiPCNA) has increased affinity for TLS polymerases, which possess a PIP (PCNA-interacting peptide) motif and ubiquitin-binding motifs (11–13). Upon fork stalling, replicative polymerases dissociate and TLS polymerases are recruited (polymerase switching).

In addition, TLS polymerases, in particular polη, are themselves phosphorylated (14–16), SUMOylated (17) and ubiquitylated (11–13) and this last post translational modification is thought to prevent their erroneous recruitment to the chromatin when they are not needed (13). Overall ubiquitylation is crucial in coordinating, controlling and activating the damage tolerance pathways. Ubiquitin and its ligases also play a fundamental role in the control of DNA replication and the signalling response to DNA damage (DNA damage response, DDR). For example, after induction of double strand breaks, histone H2A and H2AX are ubiquitylated by the E3 ligase RNF168 and they act as a recruitment platform for the repair machinery and the DNA damage checkpoint (18,19).

The recruitment of RNF168 is promoted by the activity of another E3 ligase, RNF8 (20–22), that has been shown to ubiquitylate the histone H1, a prerequisite for establishment of ubiquitylated H2A/H2AX (23). Ubiquitylation of H2A can also occur after UV irradiation in a manner dependent on the activity of the Nucleotide Excision Repair (NER), the main repair pathway that oversees the removal of UV induced DNA damage (24).

Recently UBR5/EDD1, an E3 ligase characterized by an HECT domain (25), along with TRIP12, has been shown to control the homeostasis of RNF8 and especially RNF168, by limiting the ubiquitylation of H2A/H2AX and preventing its excessive spreading from the sites of double strand breaks (26). This control mechanism has been postulated to avoid an unregulated amplification of the DDR.

UBR5 can also interact directly with a variety of players of the DDR such as p53, Chk2, an effector kinase target of the DDR, and ATMIN, a regulator of ATM (27–29). When UBR5 is depleted, G1/S and G2/M transitions are affected, leading to deficiencies in cell cycle progression, especially after DSBs (30,31). The biological importance of UBR5 is further underlined by the fact that it is frequently mutated in gastric and colon cancers and its expression is often altered in breast carcinomas (32–34). UBR5 was detected as a putative interactor of DNA polymerase η in a proteomic screen (35) and this evidence prompted us to analyse its role during the duplication of the DNA. In this work, we have unveiled a new role of UBR5 in controlling the progression of DNA replication and we have observed that when we silence UBR5 the replication fork is slowed down. This in turn leads to an increase of single stranded DNA in the cells. We have linked these problems to the mis-regulation of the UbiH2A pathway. Finally, we have discovered that when UBR5 is depleted, polη becomes toxic to the cells, possibly because of its spurious recruitment via ubiquitylated histones. These results shed light on a unified form of control of repair and replication based on the balanced homeostasis of ubiquitylated H2A.

## MATERIALS AND METHODS

### Cell culture and Plasmids

SV40-transformed MRC5SV1 and XP30RO (XP-V) cells were cultured in DMEM containing 10% FBS. The designations of the cell lines are abbreviated to MRC5 and XP30RO. XP30RO carrying eGFP tagged mutants of polη were described elsewhere (13). RFP-polη was created by sub-cloning the polη cDNA with XhoI and BamHI into pDsRedMonomer-C1 (Clontech). Plasmids carrying H2A-Flag and H2A (K13K15Q) -Flag were a kind gift from Lorenza Penengo and previously described (36). The eGFP tagged UBR5 was a kind gift from Darren Saunders. RNF8-Flag and RNF168-Flag expressing plasmids were a kind gift of Niels Mailand. UBR5 knock out cells were created by transfecting a plasmid encoding for a gRNA directed against exon 1 of UBR5 and CAS9-GFP. (Sigma Aldrich HS0000396905). After 24 h, the GFP positive cells were sorted and plated. After 15 days, single colonies were picked, expanded and screened by western blot for the presence of UBR5. Cells were treated with UV-C light at the doses indicated and harvested 6 h later. The MKR1 cell lines is derived from MRC5SV1 and has been described in (37).

### Antibodies

Antibodies used in this study included: UBR5 (#8755, CST^®^, 1:1000); anti-mouse-BrdU (Becton Dickinson, different dilutions); anti-rat-BrdU (ab6326, Abcam; 1:500); PCNA-PC10 (Cancer Research, UK, 1:1000); Vimentin (V6389, Sigma-Aldrich, 1:1000); RNF8 (ab15850, Abcam; 1:2000); RNF168 (#ABE367, Millipore; 1:500); Tubulin (clone B-5-1-2, Sigma-Aldrich); Anti-Flag M2 (F3165, Sigma-Aldrich); 53BP1 (NB100-305, Novusbio, 1:2000); Chk1-PhosphoS345 (#2341 CST^®^,1:1000); Chk1 (#2360 CST®,1:1000); Chk2-PhosphoT68 (#2661 CST^®^, 1:500); Chk2(#2662 CST®, 1:1000); TRIP12 (ab86220, Abcam; 1:500); Histone H2A(ab18255, Abcam; 1:1000); polη (custom made, raised against the peptide: VQVEQRQNPHLRNKPC, 1:1000); polη-PhosphoS601(Eurogentec,(15)); Histone H2A.X-PhosphoS139 (Upstate), RPA2 (1:1000, Millipore).

### DNA, RNA interference transfection and whole cell extracts

All RNA interference (siRNA) experiments were performed at the concentration of 20 nM using HiPerfect transfection reagent according to the manufacturer instructions (QIAGEN). All siRNA used in this study were ON-TARGETplus SMART pools (Dharmacon) with the exception of RNF8 (Life technologies) (22), TP53BP1 (Qiagen) and PCNA (Eurofins) (37). The target sequences used were: NTC-UGGUUUACAUGUCGACUAA, UGGUUUACAUGUUGUGUGA, UGGUUUACAUGUUUCUGA, UGGUUUACAUGUGUUUUCCUA; UBR5-6-CGACUUAUAUACUGGAUUA; UBR5-7- GAUUGUAGGUUACUUAGAA; UBR5-8-GAUCAAUCCUAACUGAAUU; UBR5-9-GGUCGAAGAUGUGCUACUA; UBR5 smart pool is the combination of the previous sets; USP1-7-CGACAAAGCCAACUAACGA; USP1-8-CAAAGCAGAUUAUGAGCUA; USP1-9-CAUAGUGGCAUUACAAUUA; USP1-10-GUUUGGAGUUUGAUUGUUA; TP53BP1-CAGGACAGTCTTTCCACGAAT; RNF168-5-GACACUUUCUCCACAGAUA; RNF168-6-CAAAGUAAGGCCUGGUAAA; RNF168-7-AGAAGAACAGGACAGGUUA; RNF168-8-GAAAUUCUCUCGUCAACGU; TRIP12-6-GAACACGAUGGUGCGAUA; TRIP12-7-GACAAAGACUCAUACAAUA; TRIP12-8-GCUCAUAUCGCAAAGGUUA; TRIP12-9-GGUAGUGACUCCACCCAUU. DNA plasmid transfection experiments were performed using ViaFect transfection reagent (Promega). After 72 h of knockdown treatment or transfection, the cells were directly lysed in Laemmli buffer, scraped and shortly sonicated before SDS-PAGE loading.

### DNA fibre assay

Analysis of replication fork speed and origin firing was performed essentially as in (38) after 72 h of knockdown treatment. Exponentially growing cells were pulse-labelled with 20 μM 5-chloro-2′-deoxyuridine (CldU; Sigma-Aldrich) for 20 min followed by 200 μM 5-iodo-2′-deoxyuridine (ldU; Sigma-Aldrich) for 20 min. Cells were harvested and DNA fibres spread on glass slides. After acid treatment, CldU- and IdU-labelled tracts were detected by 2 h incubation at 37°C with mouse anti-BrdU antibody (1:500, detects IdU; Becton Dickinson). Slides were fixed in 4% paraformaldehyde and incubated with rat anti-BrdU antibody (dilution 1:500, detects CldU; Abcam) overnight at room temperature. After a short blocking, the slides were incubated 2 h at 37°C with Alexa Fluor 555-conjugated goat anti-rat antibody and Alexa Fluor 488-conjugated goat anti-mouse antibody mixture (dilution 1:500 and 1:1000 respectively; both from Life Technologies). Samples were mounted in Aqua-Polymount (Polysciences, Inc., PA, USA). Fibre images were acquired using a 60× Objective on an Olympus IX71 Microscope controlled by the Metamorph Software 7.8.4 (Molecular Devices) or a 40× on a Zeiss AxioImager M2 controlled by Micromanager (39). Replicates of the experiments were acquired on the same microscope with the same settings. The lengths of CldU-(green) and IdU-(red) labelled fibres were measured with the Fiji software (40). A conversion factor of 1 μm = 2.59 kb was used as described in (41).

### FACS analysis

Cells were fixed in cold 70% ethanol at the indicated times after 30 μM bromodeoxyuridine (BrdU; Sigma-Aldrich) incubation for 30 min and stored at 4°C. DNA was denatured with 2N HCl and 0.5% Triton X-100 and then neutralized with 0.1 M sodium borate (pH 8.5). The pellet was resuspended with 0.5% Tween 20 and 1% bovine serum albumin (BSA) in phosphate-buffered saline (PBS), anti-BrdU (Becton Dickinson, Franklin Lakes, NJ, USA) was added for 1 h, followed by 1 h of anti-mouse-488 (Alexa anti-mouse 488, Molecular Probes/Invitrogen). The samples were incubated with 10 μg/ml propidium iodide and analysed on a S3 flow cytometer (Biorad, Hercules, CA, USA). The plots were analysed using FCS Express software (De Novo Software). For single propidium iodide staining, the cells were fixed as above before resuspension in PBS-0.1% Tween-20, 50 μg/ml propidium iodide, 5 μg/ml RNase A. After incubation for 15 min, the samples were analysed by FACS. Cell cycle distribution was fitted to a single cycle model with FCS Express.

### Cell fractionation

For chromatin isolation, cells were washed with PBS and incubated on ice for 5 min in buffer A (100 mM NaCl, 300 mM sucrose, 3 mM MgCl_2_, 10 mM PIPES pH 6.8, 1 mM EGTA) containing either 0.1% or 0.3% of Triton X-100 and supplemented with phosphatase inhibitors and protease inhibitors. After incubation, the Triton resistant fraction was resuspendend in Laemmli buffer. In parallel, a dish of the same cells was washed in PBS and scraped directly in Laemli buffer to prepare the whole cell extract (WCE) control.

### SDS-PAGE and western blotting

Proteins separated by SDS-PAGE were transferred to nitrocellulose membranes. After blocking with blocking buffer (Tris-buffered saline [TBS], pH 7.5, with 0.1% Tween-20 [TBST] for phospho-conjugated antibodies or phosphate-buffered saline [PBS] with 0.1% Tween-20 [PBST] containing 5% skim milk powder) for 1 h at room temperature, the membranes were incubated overnight at 4°C with the primary antibody. After incubation with secondary antibodies conjugated with HRP, the proteins were visualized using an ECL detection kit (Millipore) and the images acquired with a LAS500 system (GE Healthcare).

### Immunoprecipitation

Cells were harvested and lysed with lysis buffer (0.5% NP40, 40 mM NaCl, 50 mM Tris–HCl pH 7.5, 2 mM MgCl_2_, 1 mM *N*-ethylmaleimide (NEM), protease inhibitors cocktail (Roche), phosphatase inhibitors (Roche) and 1 ul/ml Benzonase (Novagen)) and incubated on a rotary wheel at 4°C for 30 min. After incubation, the mixture was centrifuged at 13000 rpm for 15 min at 4°C and the supernatants were collected and quantified by Bradford assay. Samples were diluted in five volumes of immunoprecipitation (IP) buffer (125 mM NaCl, 50 mM Tris–HCl pH 7.5, protease inhibitors cocktail (Roche)). Protein extracts were incubated with magnetic GFP-Trap (GFP-Trap^®^_MA, Chromotek) or appropriate antibodies coupled with magnetic beads (Dynabeads Protein G, ThermoFisher) on a rotary wheel at 4°C over night. The next day after three washes with IP Buffer, magnetic beads were re-suspended in sample buffer and samples were collected for western blot analysis.

### Immunofluorescence staining and microscopy

For native BrdU immunofluorescence staining, cells (incubated or not with 10 μM BrdU) were extracted with PBS containing 0.2% Triton X-100 for 1 min, fixed in 4% paraformaldehyde for 10 min, permeabilized with 0.5% Triton X-100 solution for 5 min, and incubated with Image-iT^®^ FX signal enhancer (Molecular Probes/Life Technologies) for 30 min. The coverslips were washed twice and incubated with primary antibodies diluted in PBS-T 3% BSA for 1 h at room temperature. After staining with secondary antibodies (Alexa Fluor; Life Technologies) for 1 h, coverslips were mounted in ProLong Gold mounting medium (Molecular Probes/Life Technologies) containing DAPI. For detecting PCNA, after fixing the cells with 4% paraformaldehyde, the cells where shortly incubated with methanol before processing for immunofluorescence as described above. All images were acquired by using a 60× Objective on an Olympus IX71 Microscope controlled by the Metamorph Software 7.8.4 (Molecular Devices) or a 40× on a Zeiss AxioImager M2 controlled by Micromanager (39). Replicates of the experiments were acquired on the same microscope with the same settings. The images were analysed by using a specific CellProfiler 2.1.1 pipeline (42).

### Proximity ligation assay

Cells were washed twice with PBS 1× and fixed with 4% paraformaldehyde for 15 min at room temperature. Cells were washed with PBS 1× and then permeabilized with 0.5% Triton X-100 for 20 min at room temperature. The cells were then blocked with 3% BSA–PBS 0.1% Tween for 1 h at 37°C and then incubated with indicated primary antibodies for two hours at 37°C. After incubation with primary antibodies, cells were washed with PLA Buffer A (Sigma) and a classic PLA reaction was performed according to the manufacturing instructions of SIGMA Duolink Kit. After the PLA reaction, cells were incubated with secondary antibodies for 30 min at room temperature, washed again with PLA buffer A (Sigma) and stained with DAPI for 2 min at room temperature. Then cells were washed twice with PLA Buffer B (Sigma) for 5 min at room temperature and one time with Buffer B 0.01× (Sigma) for 1 min at room temperature.

For protein–DNA interaction the cells, were pulsed with 10 uM EdU for 30 min before fixation in 4% PFA. After permeabilization the click-it chemistry between the incorporated EdU and biotin-azide, was performed. Cells are then, incubated with primary antibodies: anti-biotin (1:1000) and anti-UBR5 (1:1000; Abcam 70311) for 2 h at 37°C. After antibodies incubation, a classic PLA reaction was performed using SIGMA Duolink Kit according to manufacturing instructions.

### Statistical analysis

All the statistical analyses were carried out using GraphPad Prism 5 software.

## RESULTS

### UBR5 interacts with components of the replication fork

Ubiquitylation plays an important role in regulating different aspects of DNA replication, ranging from fork licensing to replication termination (43). In this context, ubiquitin controls both protein degradation and it mediates an extensive network of protein interactions. It is thus becoming clear that ubiquitin ligases are critical in allowing the correct progression of the replication fork.

**Figure 1.**
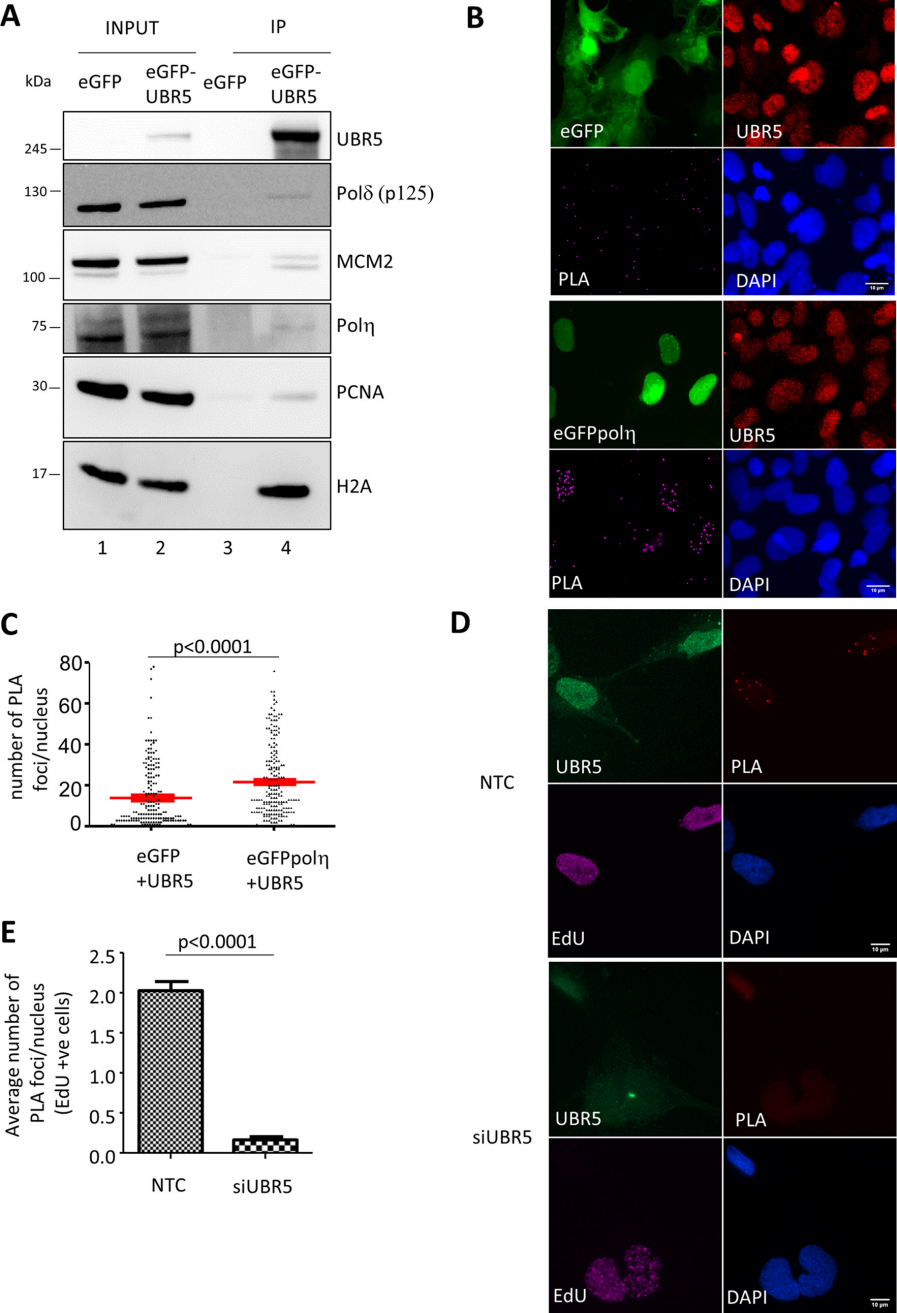
UBR5 interacts with components of the replication fork. (**A**) eGFP-UBR5 was immunoprecipitated in MRC5 cells and analysed by western blot. (**B**) Representative image of the Proximity ligation assay (PLA) used to verify the interaction between UBR5 and polη. A GFP only vector was used as negative control. (**C**) Distribution of number of PLA foci per nucleus were quantified and plotted (*n* = 3 more than 100 cells per experiment) Average ± S.E.M are indicated in red. The p value reported in the plots represent a Student's t-test. (**D**) Interaction of UBR5 with nascent DNA marked with EdU-biotin via PLA in cells silenced for UBR5 or control (NTC), the inlets show the magnification of a nucleus. Only S phase cells (EdU positive) showed PLA signal. (**E**) Average number of PLA foci per nucleus ± S.E.M. (*n* = 3 more than 100 cells per experiment).

The ubiquitin ligase UBR5 has been implicated previously in the control of DNA damage checkpoints and cell cycle transitions, but its potential role in S-phase is ill defined. In the literature, there is conflicting evidence suggesting that, in its absence, cells show either an S-phase accumulation or a complete failure to incorporate BrdU, indicating a defect in DNA replication (28,31,44). UBR5 was previously identified as a potential interactor of DNA polymerase η in a mass spectrometry screen (35), suggesting a potential role for this E3 ligase in the regulation of DNA polymerases and DNA replication. For these reasons, we began to characterize its function during DNA replication and cell cycle progression both in the absence and in the presence of DNA damage. Immunoprecipitation of eGFP tagged UBR5 allowed us to recover polη, thus confirming the original observation that UBR5 can interact with the polymerase (Figure 1A).

The interaction with polη was further tested *in vivo* with an independent approach using the Proximity Ligation Assay (Figure 1B). This assay exploits the close proximity of two antibodies directed against two antigens to start a signal amplification. An interaction between the target proteins would lead to the accumulation of a fluorescent signal detectable as discrete foci. We could detect a significant interaction and PLA signal (Figure 1B and C) between endogenous UBR5 and eGFPpolη, notably higher than the matching negative control cells expressing eGFP alone.

A more in depth analysis of the UBR5 immunoprecipitated fraction revealed that it was enriched with components of the replication machinery, including the replicating polymerase polδ, the proliferating cell nuclear antigen (PCNA), MCM2, a component of the replicative helicase and finally histone H2A (Figure 1A). This finding suggests that UBR5 could be involved in DNA replication in a more extensive role than it was previously suggested. We then endeavoured to analyse if UBR5 could directly interact with nascent DNA within a replication fork. To this end, we used a modified PLA approach where nascent DNA is marked with a short pulse of EdU that could be then biotinylated via click-IT chemistry (Figure 1D). By using an antibody against biotin we were able to detect UBR5 in the proximity of EdU, resulting in PLA foci, in S phase cells. The PLA signal was absent in cells silenced for UBR5 indicating the specificity of this approach (Figure 1D, E). EdU has been previously described as damaging to DNA (45) thus in order to minimize any potential artefacts resulting from its incorporation the cells were analysed right after the short EdU pulse.

These experimental findings suggest that UBR5 can interact with polη and other components of the replication machinery, indicating a previously undiscovered role for this E3 ligase in DNA replication.

### UBR5 controls S-phase progression

**Figure 2.**
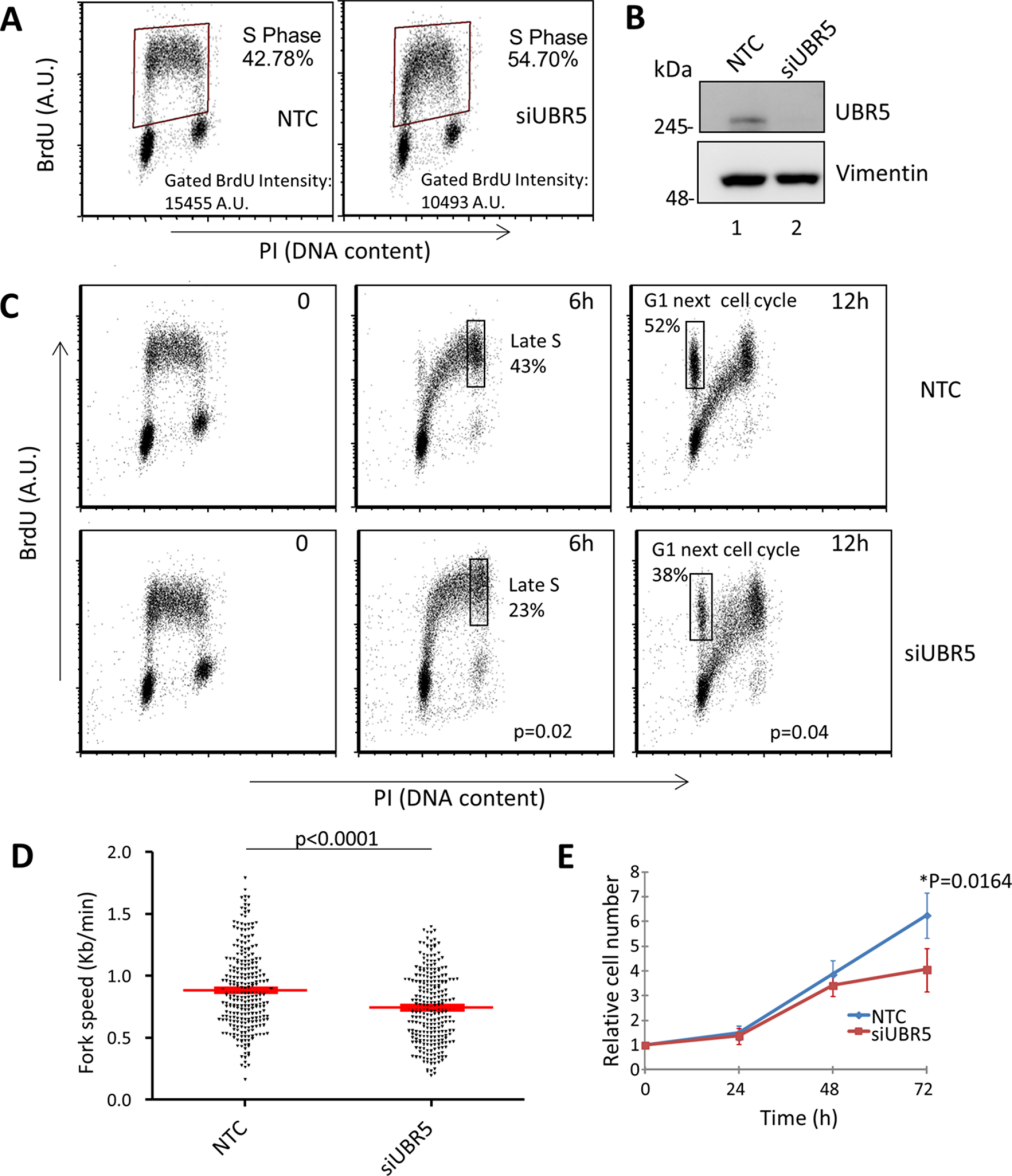
S-phase progression is slower in the absence of UBR5. (**A**) Cells were stained with an anti-BrdU antibody and counterstained with propidium iodide (PI) to monitor S phase and cell cycle distribution. The boxes represent the gating of the S-phase of the cell cycle with the mean fluorescence intensity values. (**B**) Western blot showing efficiency of UBR5 knockdown.(**C**) Representative FACS scatter plot of a BrdU pulse and chase experiment. NTC negative control and UBR5 silenced MRC5 cells were pulsed with BrdU for 30 minutes and DNA replication was followed after its removal for 6 and 12 hours. The boxes represent the average (n = 3) percentage of BrdU positive cells in the Late S/G2 phases or in the G1 of the next cell cycle at 6 and 12 hours, respectively. The p value reported in the siUBR5 plots represent a Student's t-test calculated for each gate on three independent experiments (NTC versus siUBR5). (**D**) Distribution plot of the fork speed by DNA fibre assay (*n* > 300 over three experiments) showed a decrease in fork speed in the absence of UBR5. Student's t-test p<0.0001. (**E**) UBR5 knockdown impairs cellular proliferation. After UBR5 silencing the cells were counted every 24 hours over a 72 hours time course. Plot represents mean (*n* = 5) ± S.E.M. Paired Student's t-test p<0.0164

Given these interactions, we further investigated the role of UBR5 during DNA replication. We were able to confirm an accumulation of S-phase cells when UBR5 was depleted and a concomitant reduction in BrdU intensity (BrdU fluorescence 68% of mock depleted), as previously shown in HeLa cells (Figure 2A and B) (31). We could reproduce this accumulation by silencing UBR5 with multiple independent siRNAs (Supplementary Figure S1A), excluding a possible off-target effect of the siRNA strategy. Furthermore, simultaneous over-expression of eGFP-UBR5, but not eGFP, restored the normal cell cycle distribution, as monitored by FACS (Supplementary Figure S1B). An accumulation of cells lacking UBR5 in S-phase could be explained by either a faster passage from G1 to S, a slower progression of the replicative phase or a slower transition from S to G2. So far, UBR5 has been shown to control G1/S and G2 transitions (28,30,31) but our finding of a reduced incorporation of BrdU rather suggested a slower rate of DNA synthesis during S-phase. To further investigate this, we pulsed-labelled the replicating cells with BrdU and then followed the progression of DNA replication over time after its removal. As can be seen in Figure 2C, UBR5-silenced cells show a reduced S-phase progression. Six hours after the BrdU pulse, 43% of the replicating control cells are in late S/G2 phase (Figure 2C), while only 23% of the UBR5 silenced cells had reached this cell cycle stage. This result confirms an impairment in the replication of the DNA. A slower S-phase progression could arise from a reduced speed of the replication fork. In order to assess if the replicative polymerases were slowing down when UBR5 was depleted, we performed a DNA fibre analysis, in which we monitored the length of incorporated CldU tracks. As can be seen in Figure 2D UBR5-silenced cells showed a small (15%) but reproducible decrease in fork speed. Ultimately the slow down in DNA replication impacted cell proliferation, as cells silenced for UBR5 grew slower than their matched control (Figure 2E).

### UBR5 prevents ssDNA accumulation

**Figure 3.**
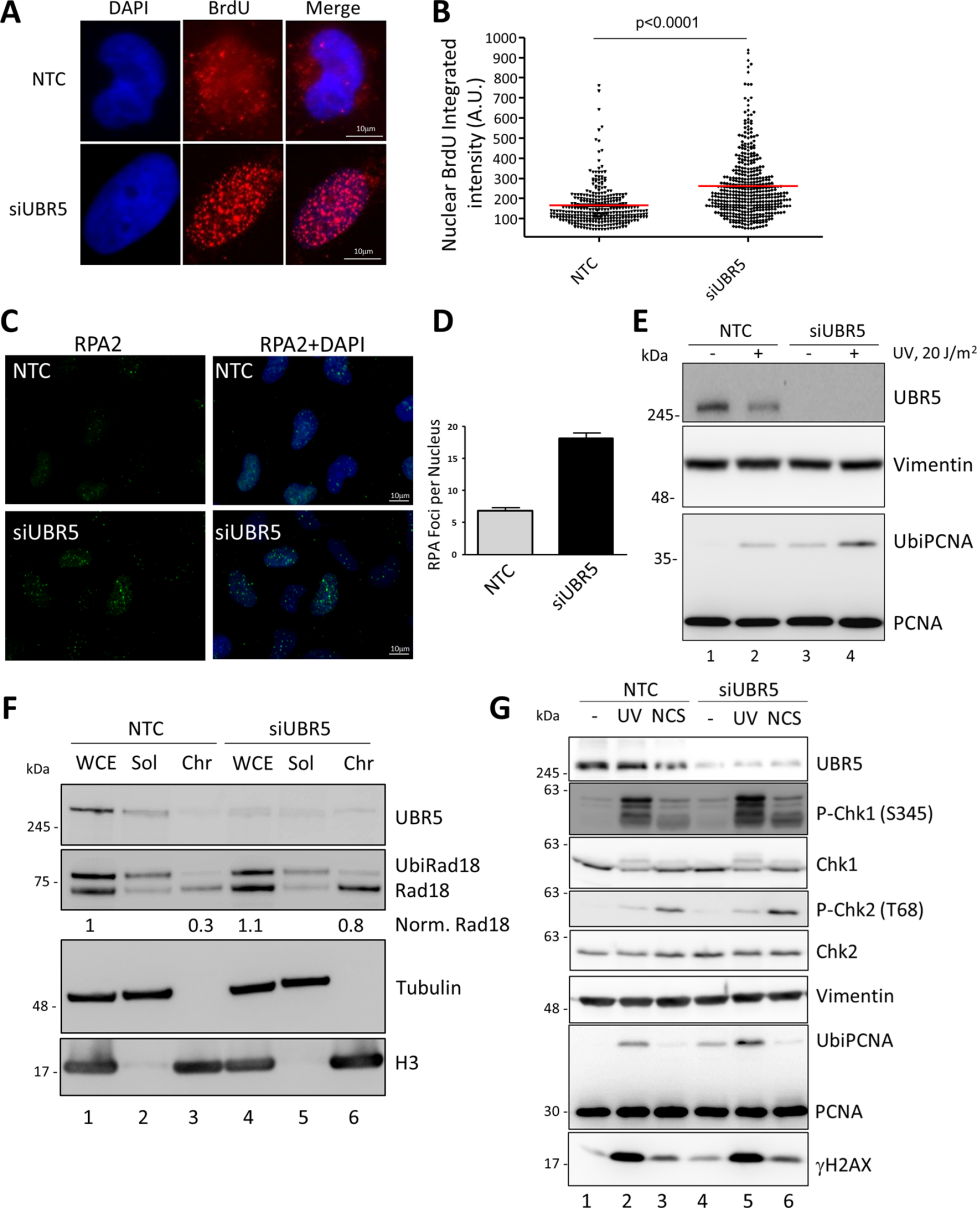
Replication defects in the absence of UBR5 lead to ssDNA and UbiPCNA accumulation. UBR5 silenced cells were incubated with BrdU for two rounds of replication (48 h). Single stranded DNA (ssDNA) accumulation was monitored by using a BrdU specific antibody in non-denaturing conditions and by measuring the integrated fluorescence intensity of the nuclei. (**A**) Representative images of BrdU in NTC and UBR5 silenced conditions. (**B**) Distribution plot of the ssDNA nuclear intensities of three experiments. Average ± S.E.M are indicated in red. Student's t-test: p<0.0001. (**C**) UBR5 silenced cells show more RPA staining than the NTC control. RPA2 staining and DAPI + RPA2 merge of representative field are presented. (**D**) Quantitation of the average RPA2 foci per nucleus (n>300 over three experiments). (**E**) Western blot analysis of whole cell extracts from NTC control and UBR5 silenced MRC5 cells. PCNA ubiquitylation was used as a marker of replication stress. (**F**) The levels of chromatin bound Rad18 were monitored after a Triton X-100 extraction from cells silenced for NTC or UBR5 (WCE: whole cell extract; Sol: Triton soluble fraction; Chr: Triton insoluble fraction). (**G**) DNA damage response analysis after UBR5 silencing in the presence of UV or Neocarzinostatin (NCS).

When the replicative polymerases are slowed down, their activity may be uncoupled from the ongoing replicating helicases. This uncoupling should generate an excess of single stranded DNA (ssDNA) ahead of the replication fork. Since we were able to detect a reduction in fork progression we decided to assess the appearance of ssDNA when UBR5 was depleted by monitoring BrdU incorporation in non-denaturing conditions. We observed an accumulation of ssDNA (Figure 3A and B) that was mirrored by an increased number of foci of the eukaryotic single strand binding protein RPA (Figure 3C and D).

Arrest of the replication machinery and the accumulation of ssDNA are features normally observed in the case of DNA damage, as generated, for example, by UV irradiation. UV light alters DNA bases and results in the formation of cyclobutane pyrimidine dimers (CPDs) and 6–4 photoproducts, two lesions that distort the DNA to different degrees (3). Replicative polymerases are not able to bypass altered templates, hence the uncoupling of helicase and polymerase activities (5). In these conditions, the E3 ligase Rad18 is recruited by ssDNA and ubiquitylates the replicating clamp PCNA (UbiPCNA). This in turn acts as recruitment platform for damage tolerance factors. We then checked the ubiquitylation status of PCNA after UV irradiation, in the presence or absence of UBR5. In unperturbed conditions hardly any PCNA is modified (Figure 3E, lane 1) and UbiPCNA can be detected only after genotoxic insult (Figure 3E, lane 2). Surprisingly, the depletion of UBR5 significantly increases the amount of UbiPCNA regardless of the presence of DNA damage (Figure 3E, lanes 3 and 4). Consistent with the appearance of UbiPCNA, we could detect more non ubiquitylated Rad18 in the Triton-X insoluble fraction, (Figure 3F, compare lane 6 with lane 3) when UBR5 was silenced, while its overall protein levels were not affected (compare lanes 4 and 1). We were able to rescue the accumulation of UbiPCNA after UBR5 silencing by concurrently overexpressing eGFP-UBR5, but not its catalytic dead allele, indicating that the E3 ligase activity is involved in this process (Supplementary Figure S1C). The replication defects were also present in a recently constructed cell line where UBR5 was knocked out by CRISPR/Cas9, further confirming its involvement in DNA replication (Supplementary Figure S2A–D). UBR5 depletion has been shown previously to cause different phenotypes depending on the p53 status of the cell line used (28,31,44). We could observe replication fork slowdown also in U2OS cells that are p53 positive (Supplementary Figure S2E/F), suggesting that the phenomenon we are observing is independent from p53.

We could still detect the accumulation of UbiPCNA in protein extracts from cells enriched and sorted in S-phase (Supplementary Figure S2G). This suggests that the increased levels of ubiquitylated PCNA could not be explained simply by the increased percentage of S phase cells after depletion of UBR5. Concomitantly with the increase in ssDNA, in the absence of DNA damage, the depletion of UBR5 elicits a weak activation of the DNA damage response with the appearance of mildly increased levels of P-Chk1 (compare lanes 1 and 4 in Figure 3G). At the same time, we could not detect an increase in the phosphorylation of H2AX or Chk2 with respect to control cells. Treatment with the ATR inhibitor VE821 did not rescue the S phase accumulation or the increase in UbiPCNA of the cells silenced for UBR5 suggesting that the phenotypes we are observing are not caused by the activation of the DDR (Supplementary Figure S2H).

After treatment with either UV or Neocarzinostatin (NCS) the phosphorylation of H2AX, Chk1 and Chk2 is present as expected, indicating that UBR5 silencing does not block the DDR in our system after damage (Figure 3G lanes 2 and 4 and lanes 3 and 6, respectively). A small increase in P-Chk1 could be observed after UV, indicating that after irradiation UBR5 depletion may lead to a stronger response of the kinase.

Overall, these results support the idea that, even in unperturbed conditions, UBR5 protects DNA replication by preventing the accumulation of ssDNA and the activation of damage tolerance mechanisms that are known to be error-prone on undamaged DNA.

### RNF168 mediates the replication defects caused by UBR5 silencing

UBR5 has been previously shown to regulate the protein levels of RNF8 and RNF168, two ubiquitin ligases that are crucial for the ubiquitylation of histone H2A after DNA damage, especially after DSB (26). We verified that silencing UBR5 increased the levels of UbiH2A (Supplementary Figure S3A), in agreement with its role in reducing the levels of RNF8 and RNF168, thus preventing its excessive accumulation and the consecutive recruitment of DDR factors to the chromatin.

Furthermore, we observed an accumulation of mono and poly-ubiquitylated species of histone H2A during the progression of S phase in synchronized cell lines (Supplementary Figure S3B). This evidence seems to support an emerging role for this modification during DNA replication (43). Interestingly, ubiquitylation of H2A has been suggested previously to regulate the replication of damaged DNA and the duplication of problematic sequences, but we could observe its accumulation also during unchallenged S phase progression.

For this reason, we wondered if the defects that we were observing upon depletion of UBR5 could be caused by a mis-regulation of RNF168. To test our hypothesis we silenced RNF168 together with UBR5 and assessed the effects of the knockdown on the ubiquitylation of both PCNA and H2A together with the cell cycle distribution.

**Figure 4.**
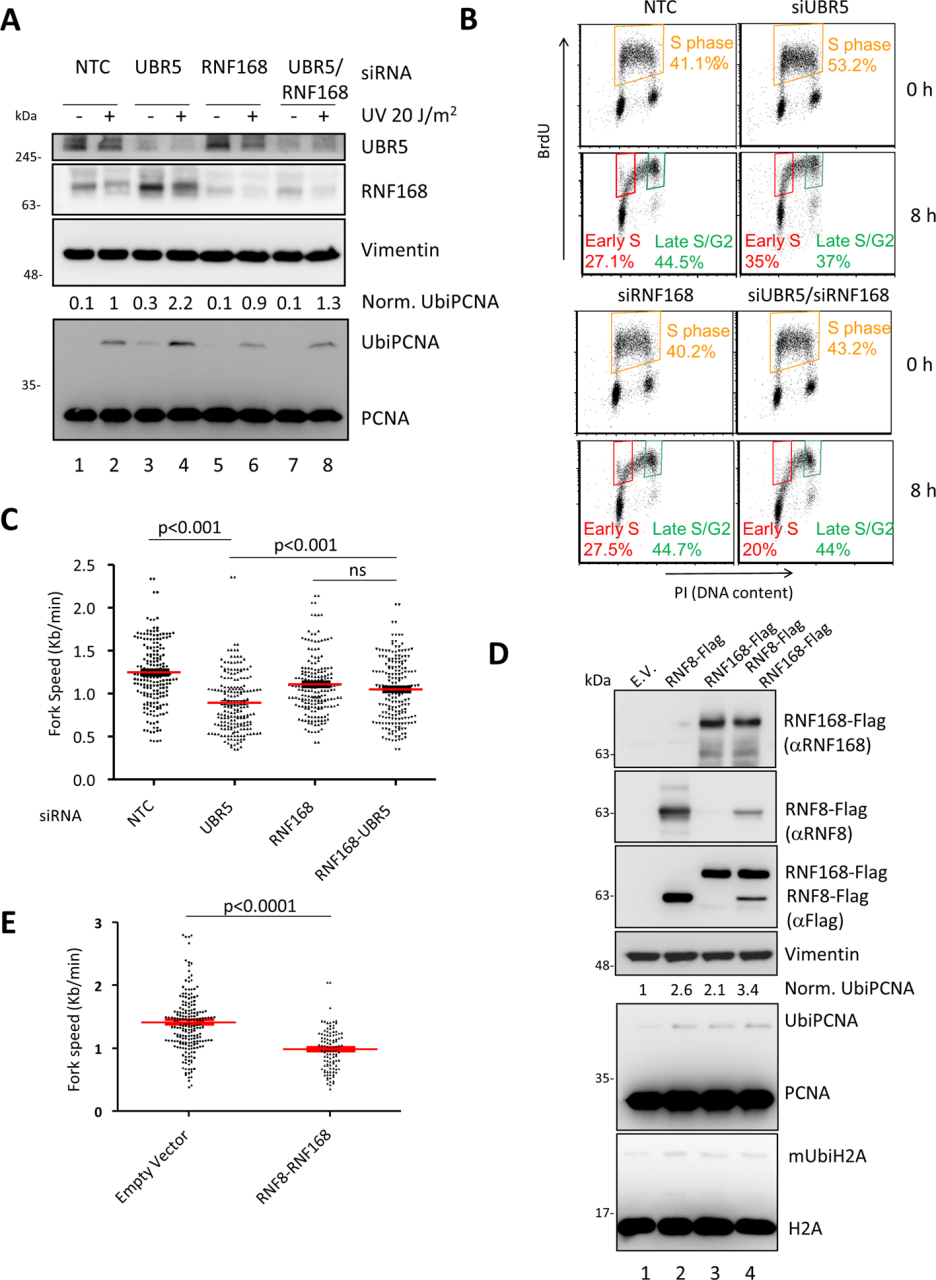
RNF168 can suppress UBR5 knockdown defects. Concomitant silencing of RNF168 and UBR5 rescues the increase of UbiPCNA (**A**), the accumulation of the cells in S-phase (**B**) and the reduction in fork speed (**C**). **(A)** Cells were transfected with different siRNAs for 72h before being harvested and immunoblotted as indicated. The normalized levels of UbiPCNA from three independent experiments are indicated on top of the PCNA blot. **(B)** Cells as in A were analysed for cell cycle distribution and S phase progression by FACS with pulse and chase experiment with BrdU (see Figure 2B). The orange gate defines the cells in S phase, while the red and green gates indicates the distribution BrdU positive cells in early S and late S/G2 respectively. **(C)** Fibre analysis of the cells silenced as in A and B. Average ± S.E.M are indicated in red. Paired Student's t-test: p<0.001. (**D**) Overexpression of RNF8, RNF168 or both induces ubiquitylation of PCNA, mimicking the phenotype of UBR5 silencing. (**E**) Fibre analysis of the cells as in D. Average ± S.E.M. are indicated in red. Paired Student's t-test: p<0.0001

As can be seen in Figure 4 simultaneous knock-down of RNF168 with UBR5 abolished the accumulation of UbiPCNA in the absence of DNA damage (Figure 4A, compare lanes 3 with 7) and reversed the modification after UV irradiation to the levels of the NTC control (Figure 4A, compare lanes 4 with 8). In these experimental conditions, we could confirm the accumulation of ubiquitin chains on H2A when UBR5 was depleted and their reduction when RNF168 was concomitantly silenced (Supplementary Figure S3C). Furthermore, after silencing RNF168, UBR5 knockdown did not lead to a delay in S-phase progression (Figure 4B bottom panel) and the resulting accumulation of cell in S phase (Figure 4B, top panel).

Consistently with this observation, the replication fork progression did not slow down when UBR5 is silenced in cells downregulated for RNF168 (Figure 4C). Interestingly silencing of RNF168 itself reduced fork speed slightly in a manner consistent with a recent report that suggests its involvement in unperturbed DNA replication (46). A similar result was obtained also by silencing RNF8 (Supplementary Figure S3D and E) indicating that the entire RNF8–RNF168 axis could be involved in regulating the effects of UBR5 depletion.

A further indication of the involvement of these E3 ligases was suggested by the fact that while their depletion was crucial for the reduction of UbiPCNA, their overexpression had the opposite effect leading to its accumulation (Figure 4D) and also resulted in a decrease in fork speed (Figure 4E).

RNF168 protein levels are also regulated by TRIP12, another E3 ligase. Its depletion led to an increase in UbiPCNA similar to the one observed by the silencing of UBR5 (Supplementary Figure S4A).

Altogether, these experiments show that the defects in replication caused by the lack of UBR5 are mediated by the RNF168 axis and they suggest that its accumulation, and possibly activity, can be detrimental to the progression of DNA replication.

### UbiH2A causes replication problems

**Figure 5.**
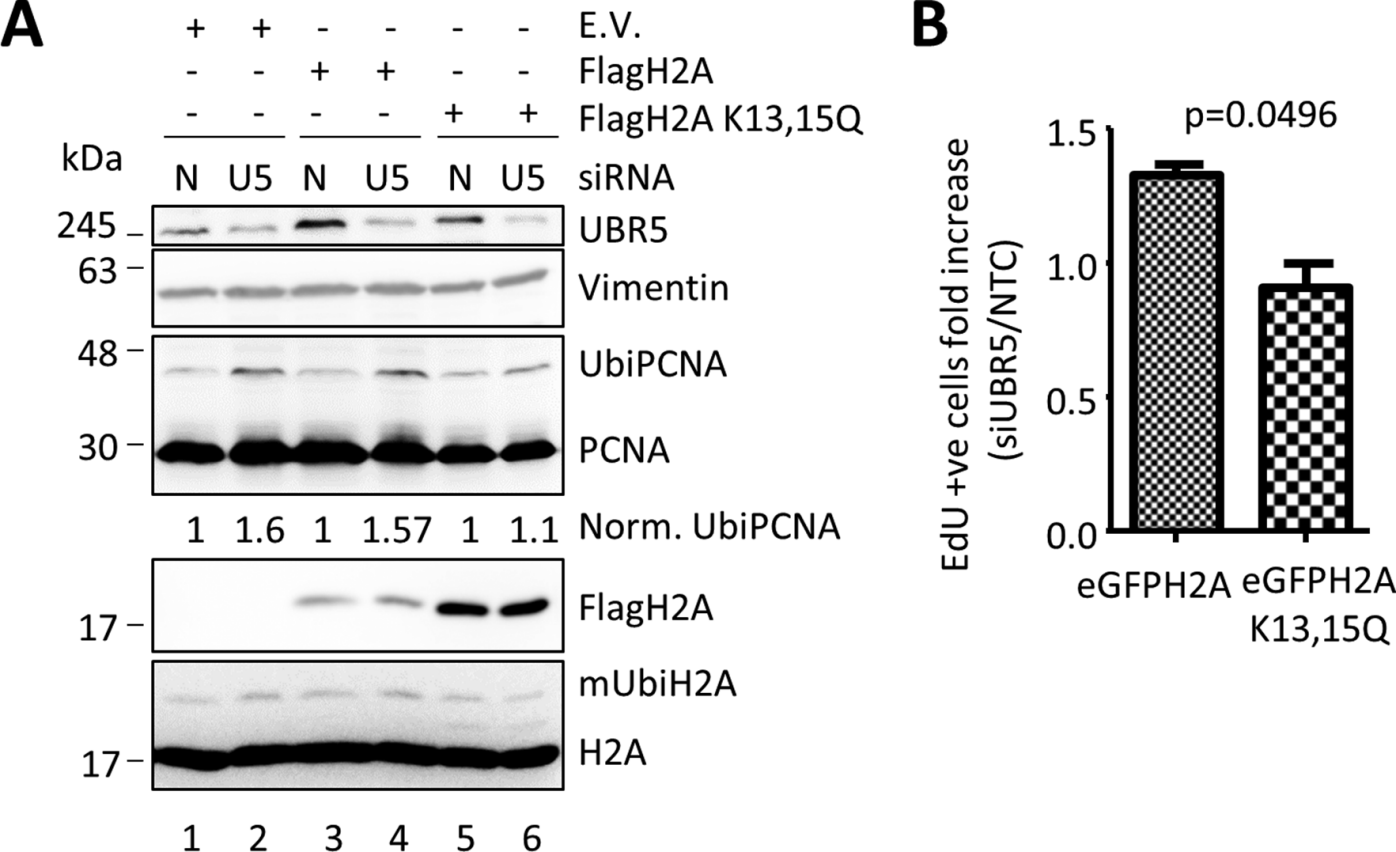
Replication defects are caused by ubiquitylation of H2A. A mutant of H2A that cannot be ubiquitylated by RNF168 is not sensitive to replication problems caused by UBR5 depletion. (**A**) MRC5 cells were transfected with either empty vector (E.V.), Flag-H2A or Flag-H2AK13,15Q before UBR5 silencing. After 72 h, the cells were collected and processed for Western Blotting. The numbers on the blot represent the normalized intensity of the UbiPCNA, corrected for the internal loading control. (**B**) Overexpression of H2AK13,15Q blocks the accumulation of cells in S phase in the absence of UBR5. EdU positive nuclei were scored in the presence of either overexpressed eGFPH2A or eGFPH2AK13,15Q in the presence or the absence of UBR5. Number of EdU positive nuclei in siUBR5 cells were normalized on their matched NTC control. (n=2 more than 100 cells per experiment).

RNF8 and RNF168 have been shown to control the ubiquitylation of H2A (47). Indeed, an alteration in the steady state levels of the two ligases results in accumulation of UbiH2A on the chromatin (26). To verify that the replication defects detected when UBR5 was silenced were due to a misregulation of UbiH2A, we expressed, in the cells, an allele of H2A that is mutated in the two crucial lysines (K13 and K15) that have been demonstrated to be the target of RNF168 mediated ubiquitylation (36,48). We reasoned that by overexpressing this mutant form of H2A we could remove the target of RNF168 and rescue the phenotypes observed after UBR5 knockdown. As can be seen in Figure 5A, expression of H2AK13,15Q decreases the amount of UbiH2A (by ∼40%, compare lane 6 versus lanes 2 and 4) and reverses the increase in PCNA ubiquitylation when UBR5 is silenced (compare lane 6 with lanes 2 and 4). Similarly, the expression of the mutated histone prevented the increase of cells in S phase when UBR5 was downregulated, as monitored by EdU incorporation (Figure 5B). Altogether, this hints to ubiquitylated H2A as the ultimate cause of the replication problems induced by the absence of UBR5.

The presence of UbiH2A increases its affinity for ubiquitin binding proteins and its accumulation could lead to a spurious recruitment of DDR factors to the chromatin, as can be exemplified by the formation of 53BP1 foci in the absence of DNA damage when UBR5 is knocked down (26). An untimely recruitment of DDR factors could be the underlying cause of replication problems, possibly due to the collision of different molecular machineries in the delicate S-phase of the cell cycle. If this were the case, removal of a factor like 53BP1, would restore normal replication like the depletion of RNF8 and RNF168. Interestingly, removal of 53BP1, in conjunction with UBR5, did not abrogate either the accumulation of UbiPCNA (Supplementary Figure S3B) or the increase in the number of cells in S-phase as monitored by FACS (Supplementary Figure S3C). Thus, we can exclude the possibility that the phenotypes we are observing are the result of an accumulation of 53BP1 that impedes replication fork progression.

### UBR5 protects the cells from polη mediated cell death

**Figure 6.**
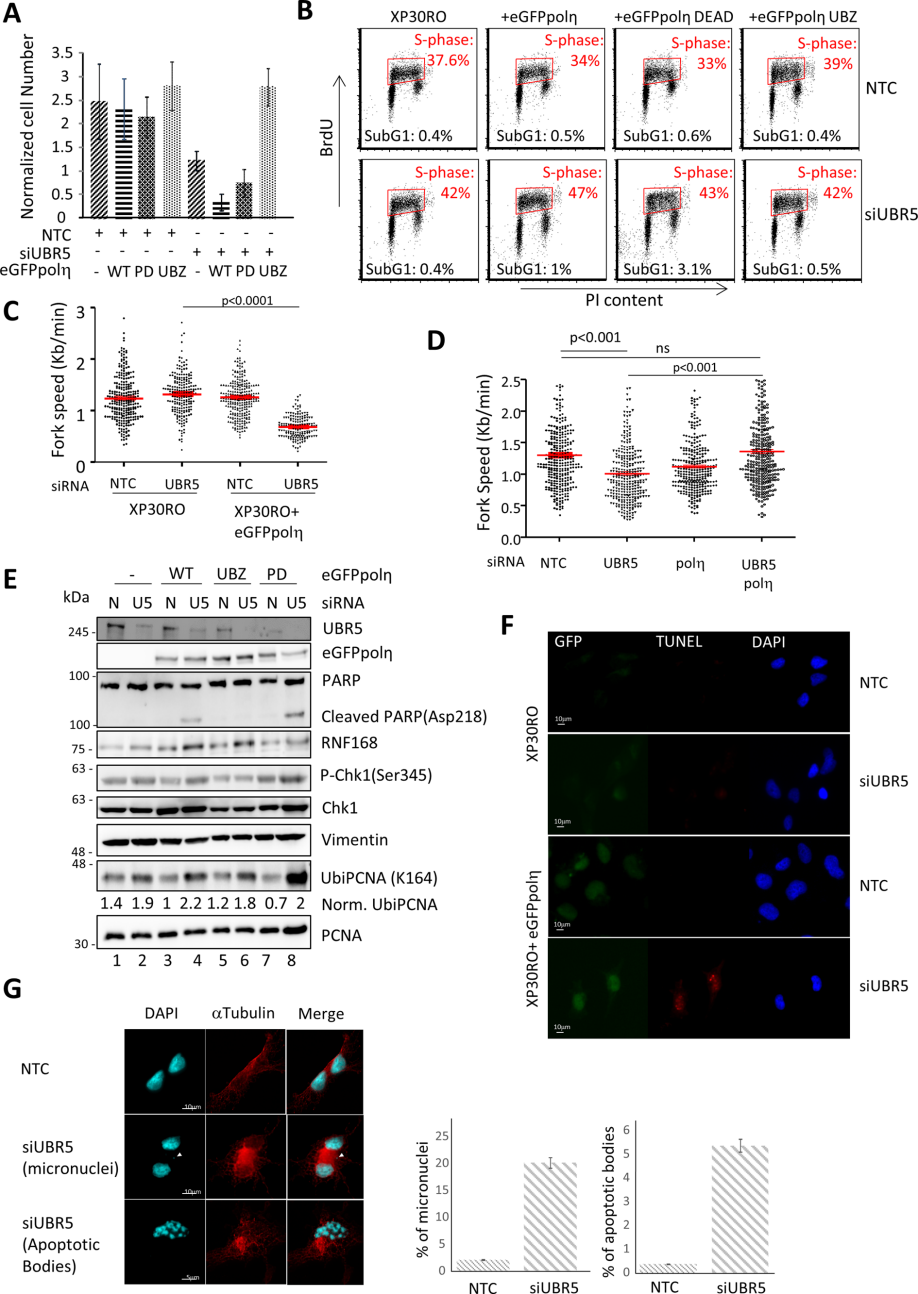
Polη interferes with DNA replication in absence of UBR5. The presence of DNA polymerase η causes replication fork slow down and genetic instability in absence of UBR5. (**A**) The growth of XP30RO (polη deficient cells) and matching restored cell lines expressing eGFPpolη, either WT, mutated in its catalytic (PD) or UBZ domains was monitored over the course of 72 h. The presence of a functional polη results in a decrease in cell number. The plot represents the mean of three experiments ± S.E.M. where the cell number was normalized on the number of cell seeded at the beginning of the experiment. (**B**) FACS analysis of XP30RO and matching restored cell lines expressing eGFPpolη, either WT or mutated in various motifs show an increase in the sub-G1 (population of the cells expressing eGFPpolη. All the plots show 10000 events. The average number of cells in Sub-G1 and in S-phase derived from two independent experiments are included in each plot. (**C**) XP30RO cells lacking polη do not slow down the replication fork speed in the absence of UBR5 (n=2 more than 100 fibres per condition per experiment) Average ± S.E.M are indicated in red, p value represents a Paired Student's t-test. (**D**) Silencing of polη rescues the fork speed reduction in MRC5 cells knocked down for UBR5 (n=3 more than 100 fibres per condition per experiment). (**E**) Western blot analysis showing increased PARP1 cleavage when WT polη is present and UBR5 is silenced. (**F**) TUNEL assay of XP30RO and matched complemented cell lines. (**G**) UBR5 was silenced in MRC5 cells for 72 h before treatment with cytochalasin-B for 24h. After fixation, the cells were stained for DAPI and αTubulin. Micronuclei positive bi-nucleated cells (n>900 between two experiments) and cells showing apoptotic bodies were scored.

An increase in UbiH2A could provide a scaffold for the recruitment of factors other than those involved in DSB repair. The defects we observe in the absence of UBR5 are mostly related to the S-phase of the cell cycle, so we investigated if enzymes involved in ensuring the correct progression of DNA replication could account for its phenotypes. Translesion synthesis polymerases are able to bind ubiquitin via their ubiquitin binding motifs. Among them, the most characterized is DNA polymerase η, which is responsible for the error-free bypass of CPDs. We were surprised to find that, when UBR5 was depleted, cells lacking polη (XP30RO) grew substantially better than their complemented counterpart, although a moderate (50%) decrease in duplication potential could be still observed in XP30RO cells, silenced for UBR5, when compared with the scrambled control (Figure 6A).

Cell cycle analysis revealed that the accumulation in S-phase was reduced in XP30RO cells silenced for UBR5 when compared with the complemented cell line (Figure 6B). We also observed an increase in the population of cells showing sub-G1 DNA content in the restored cell line, suggestive of an increased proportion of dying cells (Figure 6B). This accumulation was present also when polη was catalytically inactive but disappeared when its UBZ was mutated, indicating that the effect on the progression of DNA replication required polη to bind to ubiquitin (Figure 6B). In line with this, we found that XP30RO cells did not show a slowing down of the replication fork when UBR5 was depleted (Figure 6C).This evidence suggests that polη could be a source of replication problems when UBR5 was depleted, but also indicated that, in its absence (e.g. XP30RO cells), other factors could be detrimental to cell duplication or viability.

It is relevant to know that XP30RO complemented cells (XP30RO+eGFPpolη) moderately overexpress polη at two to three times the physiological levels of MRC5 cells. Despite this, we can appreciate that the overexpression of the polymerase does not cause problems, as the NTC control (Figure 6C) does not show any impairment in replication fork progression. Only when UBR5 is downregulated polη becomes an obstacle for the replication fork. In addition, the reduction in fork speed observed in the absence of UBR5, in MRC5 cells, is rescued by the depletion of polη (Figure 6D) indicating that the polymerase is negatively affecting replication fork progression when UBR5 is downregulated. It is worth noting that also MRC5 cells show an increase in the sub-G1 population, albeit to a lower extent, indicating that UBR5 silencing is causing cell death in cells that are not overexpressing polη (Supplementary Figure S5A).

The decrease in cell number observed when polη was expressed in XP30RO cells, after UBR5 silencing, was mirrored by a stronger accumulation of UbiPCNA in the WT and PD cell lines and by the appearance of the cleaved form of PARP1, indicating that the cells were undergoing apoptosis (Figure 6E), as confirmed by a TUNEL assay (Figure 6F). All the stable cell lines showed comparable accumulation of K63 ubiquitin chains in histone acid extracts when UBR5 was depleted (Supplementary Figure S5B).

Replication defects leading to cell death could be a sign of genome instability. For this reason, we monitored the appearance of micronuclei in MRC5 cells, that are not overexpressing polη, depleted by UBR5 in order to evaluate if the replication problems could be ultimately resolved. As can be observed in Figure 6G, the silencing of the E3 ligase led to a marked increase in the number of bi-nucleated cells showing micronuclei and, consistent with the previous observation that UBR5 protects from apoptosis, we observed a number of cells characterized by apoptotic bodies. Both populations were absent in the control treated cells.

UBR5 silencing results in increased PCNA ubiquitination, and this could be the event that generates a toxic effect by spuriously recruiting polη. A first indication that this was not the case was the fact that an artificial increase of UbiPCNA, via depletion of its deubiquitinating enzyme, USP1 (37,49), did not lead to an impediment in the growth of the cells or accumulation of cells in S-phase (Supplementary Figure S5C/D). Our hypothesis that UbiPCNA was not responsible for the phenotypes observed, was further strengthened when we exploited a cellular background where PCNA could not ubiquitylated. We achieved this by using a stable cell line expressing a PCNA K164R mutant that was resistant to a siRNA targeting endogenous PCNA (37). In these cells, upon knockdown of endogenous PCNA, we could still observe slowing down of the replication fork when UBR5 was depleted (Supplementary Figure S5E), despite the absence of PCNA ubiquitylation (see Supplementary Figure S5F, lane 4). Together these two pieces of data suggest that the appearance of UbiPCNA is not the cause of the replication problems, when UBR5 is silenced, but it is rather a consequence of the slow down of the replication fork and the consequent build-up of ssDNA (Figure 3A–C).

### UbiH2A recruits polη to the chromatin

**Figure 7.**
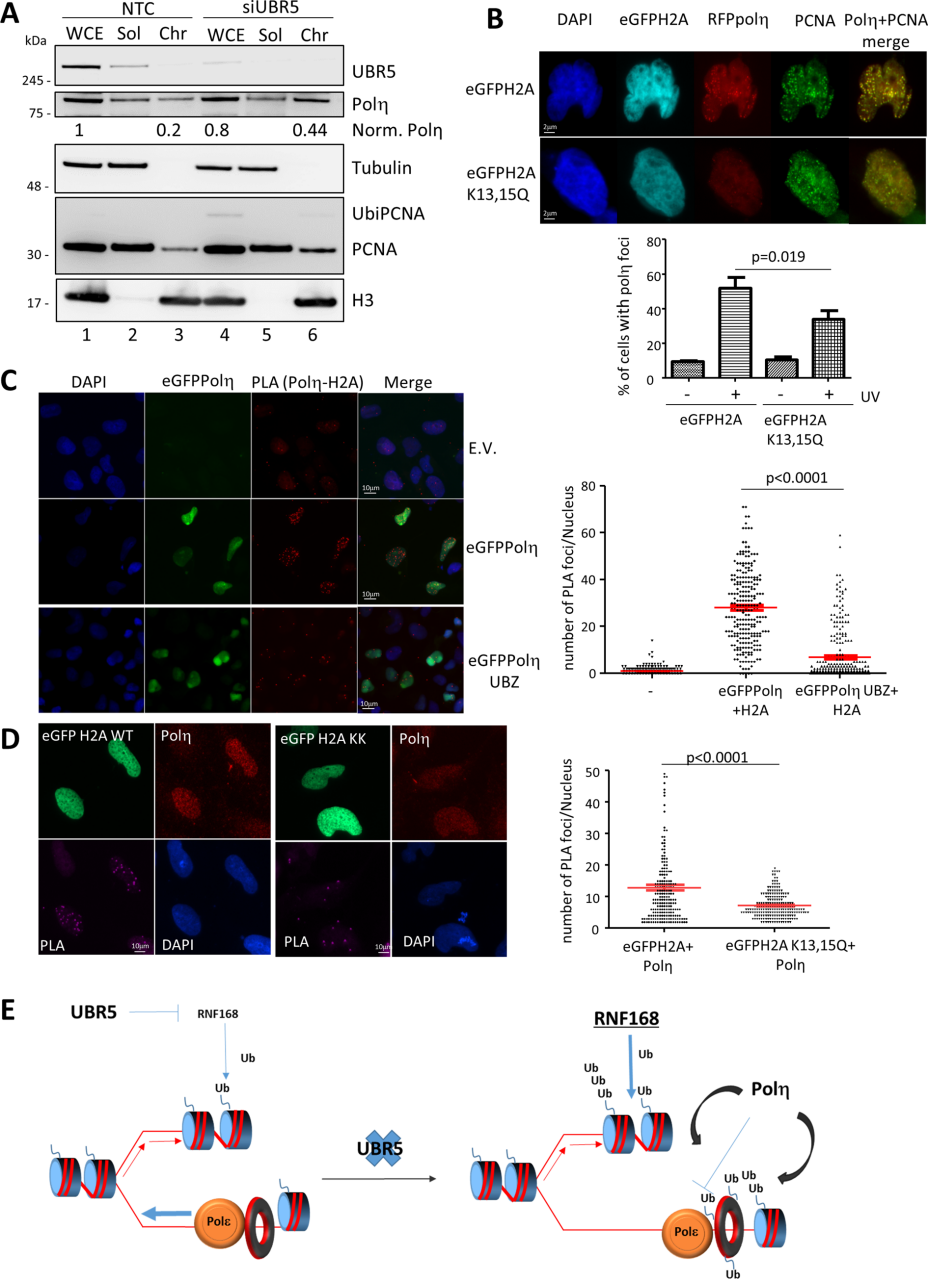
UBR5 and UbiH2A control recruitment of polη to the chromatin. (**A**) Western blot analysis of the Triton X-100 resistant chromatin fraction of polη from MRC5 cells silenced for NTC control or UBR5 in the absence of DNA damage (WCE: whole cell extract; Sol: Triton soluble fraction; Chr: Triton insoluble fraction). Normalized levels of polη are indicated in red for the WCE and Chr fractions (**B**) H2A can control the recruitment of polη to replication foci. Representative image of RFP-polη foci after UV in the presence of eGFP-H2A or eGFP-H2AK13,15Q. Indirect immunostaining of PCNA was used as positive control. The plot shows the mean of percentage of the the number of eGFP positive cells showing RFPpolη foci ± S.E.M. (n=3), the p value represents a Paired Student's t-test. (**C**) PLA analysis between eGFPpolη and H2A shows that the interaction is mediated by polη’s UBZ. eGFPpolη and one allele mutated in the UBZ were transfected in MRC5 cells and interaction between H2A and polη was analysed via PLA. Representative image on the left panel and quantitation on the right panel. The plot represents the distribution of number of PLA foci per nucleus in each sample (n=2 more than 100 cells per experiment). (**D**) PLA analysis of the interaction between polη and ubiquitylated H2A. eGFP-H2A and eGFP-H2Ak13,15Q were transfected in MRC5 cells and their interaction with endogenous polη analysed via PLA. Representative image on the left panel and quantitation on the right panel. The plot represents the distribution of number of PLA foci per nucleus in each sample (n=2 more than 100 cells per experiment). (**E**) UBR5 regulates the protein levels of RNF168 ultimately controlling the amount of ubiquitylated H2A. When these UbiH2A levels are maintained, DNA replication can progress unhindered (left panel). In the absence of UBR5, RNF8 and RNF168 accumulate, and eventually enough UbiH2A is established to result in the unregulated recruitment of polη, via its UBZ domain (right panel), and the subsequent reduction in replication fork progression. For clarity, only effects on the leading strand are shown, as these are likely to affect the rate of fork progression.

The data presented suggests that UBR5 might protect the cells from a toxic activity of polη. We reasoned that in order to perform any activity the polymerase should be recruited onto the chromatin and that, in the absence of UBR5, we should be able to detect this accumulation. In untreated conditions, only ∼23% of total endogenous polη was present in the TritonX-100 insoluble fraction of MRC5 cell extracts (Figure 7A, lane 3) but this fraction was more than doubled to 55% if UBR5 is silenced (Figure 7A, lane 6).

Ubiquitylated PCNA is thought to be the main ubiquitylated protein that binds the UBZ of polη but some experimental evidence suggests that UbiPCNA is not strictly required to recruit the polymerase to the chromatin, even though the UBZ of polη was absolutely required (discussed in Gohler *et al.*, 2011). We speculated that another ubiquitylated target could help polη in this crucial step. UbiH2A has been previously reported to accumulate after UV damage (24). A role for UbiH2A in controlling polη recruitment could explain why its de-regulation, when UBR5 is depleted, is detrimental to the cells in the presence of the polymerase. Expression of H2AK13,15Q significantly reduced the accumulation of polη into replication foci after UV (Figure 7B) suggesting that ubiquitylated H2A could help in the relocation of the polymerase to replication factories. Concomitantly the UV dependent phosphorylation of chromatin-bound polη on Serine 601 (15) was reduced in the presence of the mutated histone suggesting that, to some extent, UbiH2A targets polη to the chromatin where it can be phosphorylated by ATR (Supplementary Figure S5G). The results presented so far point to a role of ubiquitylated H2A in the recruitment of polη to the chromatin and to replication factories. This could be explained by a direct interaction between polη and UbiH2A, possibly mediated by the polymerase UBZ. This hypothesis was supported by finding that we could detect polη in the H2A immunoprecipitated fraction (Supplementary Figure S5H and I) and their proximity in the nucleus was visualized further via PLA assay (Figure 7C). Immunoprecipitation and proximity ligation showed a stronger interaction when the UBZ of polη was intact (Figure 7C and Supplementary Figure S5H). In addition, the interaction between polη and H2A was reduced if H2A was mutated in K13 and 15, suggesting that the modification on those two residues is important for polη docking (Figure 7C and D and Supplementary Figure S5I). Once again, it is possible to speculate that UbiPCNA could mediate the proximity of H2A and polη, despite our analysis being performed in undamaged cells where the amount of such modification is limited. To overcome this issue, we confirmed the PLA assay in the cellular background where PCNA ubiquitylation was blocked (Supplementary Figure S6A–C), indicating that UbiPCNA is not required for H2A and polη interaction.

## DISCUSSION

Ubiquitylation plays a crucial role in the regulation of DNA damage response, damage tolerance and DNA replication as it helps bridge those DNA transitions and coordinate complex cellular responses.

Ubiquitylation of H2A has been found to control gene transcription and the recruitment of DNA repair factor to double strand breaks (26), but its role in DNA replication is largely unknown. At the same time, little is known about the roles of the E3 ligases that control the homeostasis of H2A ubiquitylation and how unbalancing this delicate equilibrium could affect the duplication of the genome. Here we show that ubiquitylated H2A increases during the S phase (Supplementary Figure S3B) and one of the E3 ligases responsible for its regulation, UBR5, interacts with elements of the replication fork. Furthermore we show that, UBR5, which was previously found to be involved in DSB repair, participates in the control of the progression of the replication fork.

In the absence of UBR5, cells accumulate in S phase due to a slower fork progression. UBR5 has been previously related to problems with the progression through S-phase but this was linked to its control of Cdc25a, E2F1 and p21-p27, leading to an S-phase accumulation as a result of a failure to inhibit CDK2/cyclin complexes (31). Here we show that the problems arising in the absence of UBR5 are directly correlated with a role of the E3 ligase in controlling the state of the chromatin at the replication fork. Concomitant with the slowing down of replicative polymerases, we observe an accumulation of ssDNA covered by RPA and the ubiquitylation of PCNA, a trigger for the activation of the DNA damage tolerance systems. We further show that the effects of UBR5 depletion work via the RNF168 and UbiH2A pathway. While this manuscript was in preparation, the groups of Penengo and Lopes found that UbiH2A was important for unperturbed DNA replication and limiting its amount, by depleting RNF168, resulted in replication stress (46). Our results complement their findings indicating that also its upregulation needs to be tightly regulated to allow normal DNA replication. At this point, an important question is what is the role of ubiquitylated H2A during replication. We show that this modification is important in helping the recruitment of DNA polymerase η after UV and it is needed for its phosphorylation on S601, a prerequisite for efficient TLS (15). In the presence of DNA damage, this form of recruitment is beneficial for the establishment of a successful damage bypass, as in the absence of K13 and K15 of H2A, polη is not efficiently phosphorylated nor efficiently recruited to replication factories. The close relationship between polη and H2A is further exemplified by their interaction *in vivo* in a manner that is dependent on polη UBZ and lysines 13 and 15 of H2A.

When damage is not present, TLS has to be tightly controlled in order to avoid undesired mutagenesis.

What we show is that, in unchallenged conditions, UBR5 downregulation results in increased levels of UbiH2A, allowing polη to be recruited more extensively to the chromatin (Figure 7A). During S-phase, polη is then in close proximity with the replication fork and it has unregulated access to the replication machinery disrupting its progression (Figure 7E). The catalytic domain of polη is only partially required to observe the replication defects, so we tend to favour a model where the polymerase acts both as a steric impediment and its activity is somehow detrimental to the cell. We have shown previously that ubiquitylation of PCNA is required for efficient TLS (37), but its depletion, by treatment with the proteasome inhibitor epoxomicin, does not impair polη foci formation (50), indicating that this step and the polymerase initial recruitment to the chromatin is independent from PCNA modification. Furthermore, phosphorylation of polη on S601, which occurs only on the chromatin, requires the UBZ domain of polη and Rad18 but not PCNA ubiquitylation (15). These lines of evidence, in addition to the defects we show in polη recruitment and S601 phosphorylation after UV damage in the presence of H2A K13,15Q led us to suggest that UbiH2A could play a role in the initial recruitment of the polymerase to replication factories before polη can be engaged in TLS via its interaction with UbiPCNA.

In the absence of a tight control of UbiH2A, a loading platform for polη and other repair factors, could form, resulting in replication problems (Figure 7E).

Erroneous recruitment of TLS polymerase has always been considered as a potential source of mutagenesis (5). Here, we show that polη could even block the normal progression of the replication fork. It is interesting to note that overexpression of polη does not show such a phenotype (Figure 6C) (50–52), when UbiH2A homeostasis is controlled. This suggests that the correct recruitment of TLS proteins and their regulation is more important than their overall protein amount.

One of the dogmas concerning TLS polymerases has always been the need for stalling of the replicating polymerases in order to execute a polymerase switch. This paradigm has been established by convincing *in vitro* data generated in bacterial and eukaryotic systems. In both cases TLS polymerases, either PolIV in *Escherichia coli* or polη were not able to access the replication fork while PolIII or Polδ, respectively, were actively replicating (53,54). Our *in vivo* data suggests that under certain conditions, a TLS polymerase can override this form of control and it can perturb the progression of the replicating polymerase. This is consistent with another observation where polκ was able to perturb fork progression in the presence of an excess of UbiPCNA caused by the depletion of USP1 (55). In those experimental conditions, only polκ, but not polη, was found to be toxic to the cells. Interestingly, no alteration in the overall progression of the S-phase was reported. Indeed, we could not detect any alteration in cell cycle progression in our cells when USP1 was silenced. Differently from polκ, whose aberrant recruitment was linked to a ubiquitylated substrate known for its role in TLS (UbiPCNA), our work points to a new role for ubiquitylated H2A as an interaction partner of polη. Ultimately, an alteration of UbiH2A levels appears to have a stronger phenotype on DNA replication than the one reported for USP1.

We have previously shown that the localization of TLS polymerases η and ι is extremely dynamic, even in the presence of DNA damage, and their interaction with DNA is transient and labile (50). This supported the notion that TLS polymerases could engage the DNA only in the presence of a potential substrate and then they could be released quickly once they had accomplished damage bypass. Indeed, we found that UbiPCNA was used as a stabilizing factor that could slow down the movement of polymerases into/out of replication foci (50). It is possible to argue that, as in the case of polκ, the increase in UbiPCNA, resulting from UBR5 depletion, could act as the canonical loading platform for polη instead of UbiH2A. However, USP1 depleted cells, which show a substantial increase of UbiPCNA, have neither a replication nor a growth problem in our experimental system. In addition, the slowing down of the replication fork, when UBR5 was silenced, persisted even in cells where PCNA cannot be ubiquitylated, indicating that UbiPCNA *per se* is not sufficient to explain the phenotypes that we observed.

In conclusion, we show here that an interaction between UbiH2A and polη is important for regulating the recruitment of polη to replication factories and its subsequent phosphorylation. This new step in controlling translesion synthesis needs to be precisely modulated as an alteration of the correct homeostasis of UbiH2A could be critical not only for an efficient DNA damage response but also for the maintenance of ongoing DNA replication.

## Supplementary Material

gkz824_Supplemental_File
