# Supplementary material for: UBR5 interacts with the replication fork and protects DNA replication from DNA polymerase η toxicity
Source: Nucleic Acids Res. 2019 Oct 5;47(21):11268–83. doi: 10.1093/nar/gkz824 (PMC6868395; doi:10.1093/nar/gkz824)
Supplement: gkz824_Supplemental_File [file gkz824_supplemental_file.pdf]

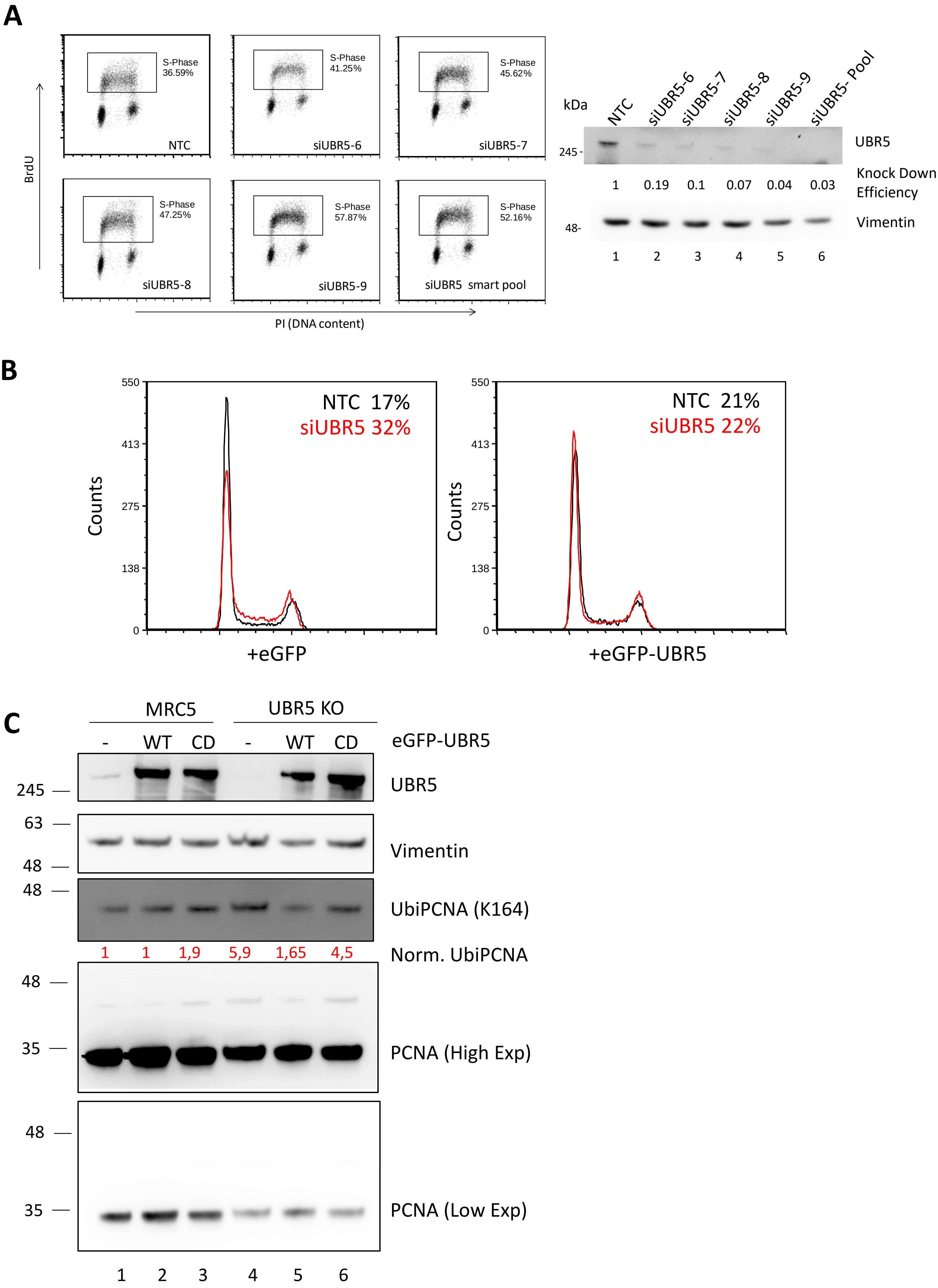

Figure-S1_Cipolla

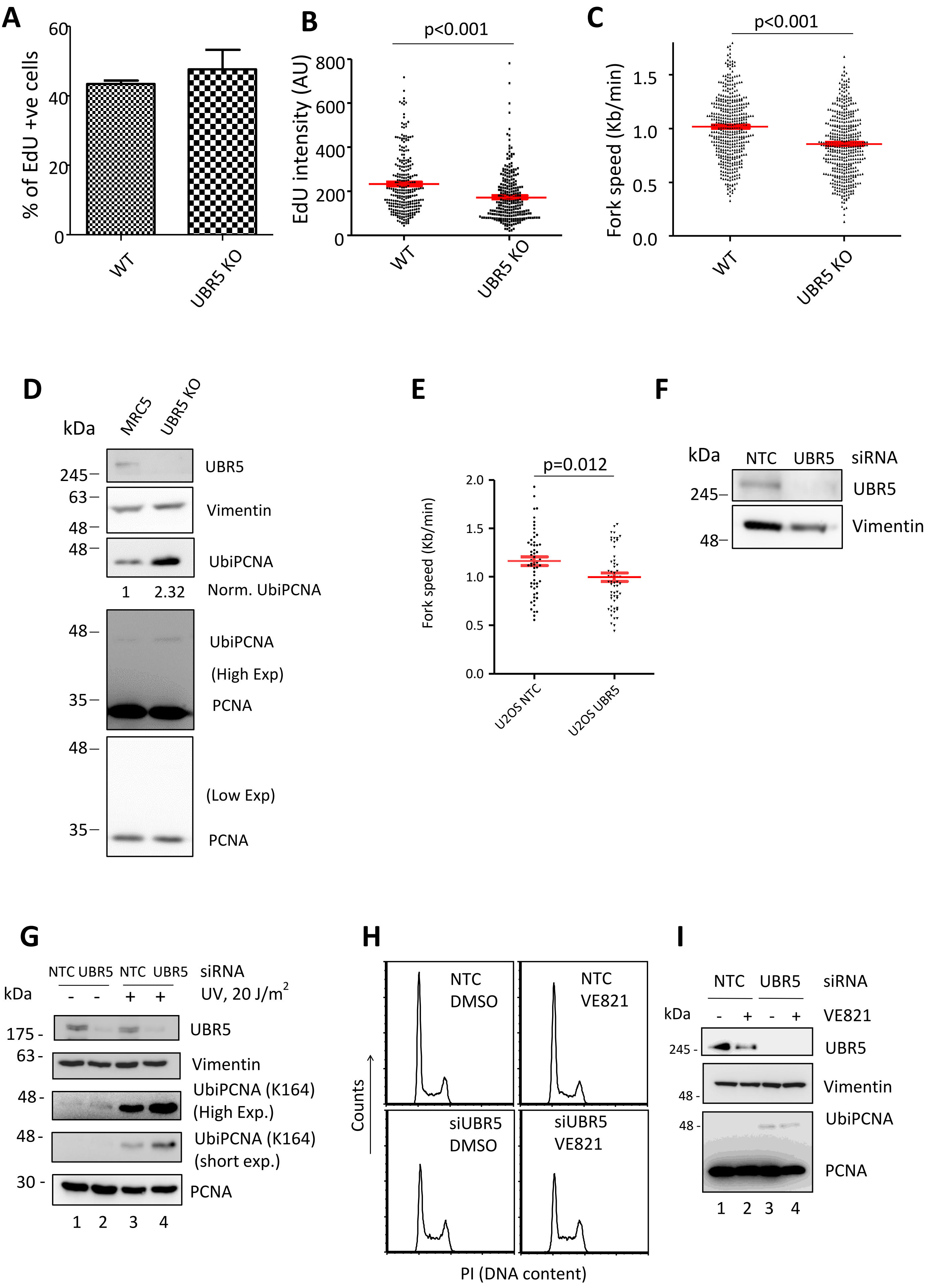

Figure-S2_Cipolla

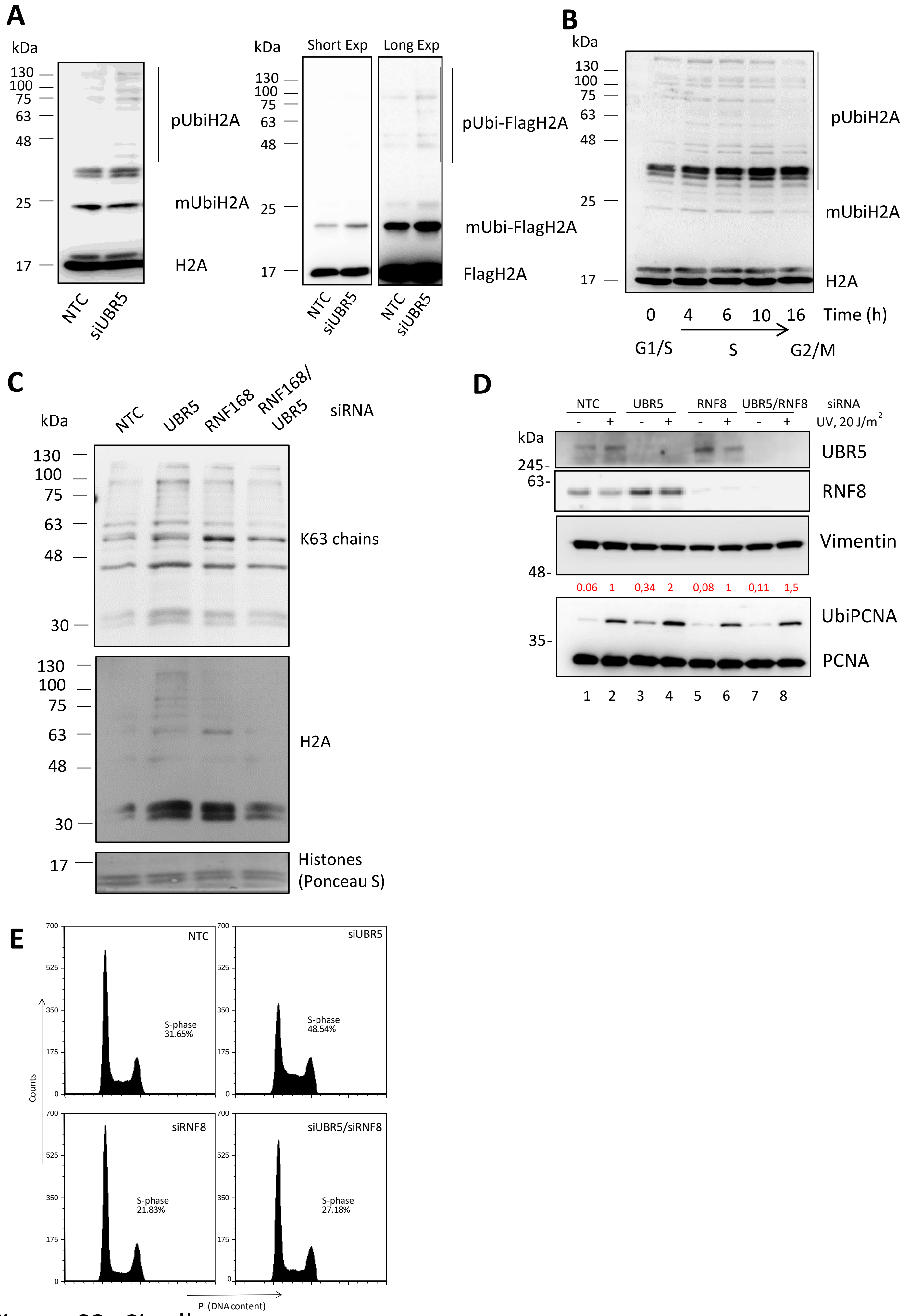

Figure-S3_Cipolla

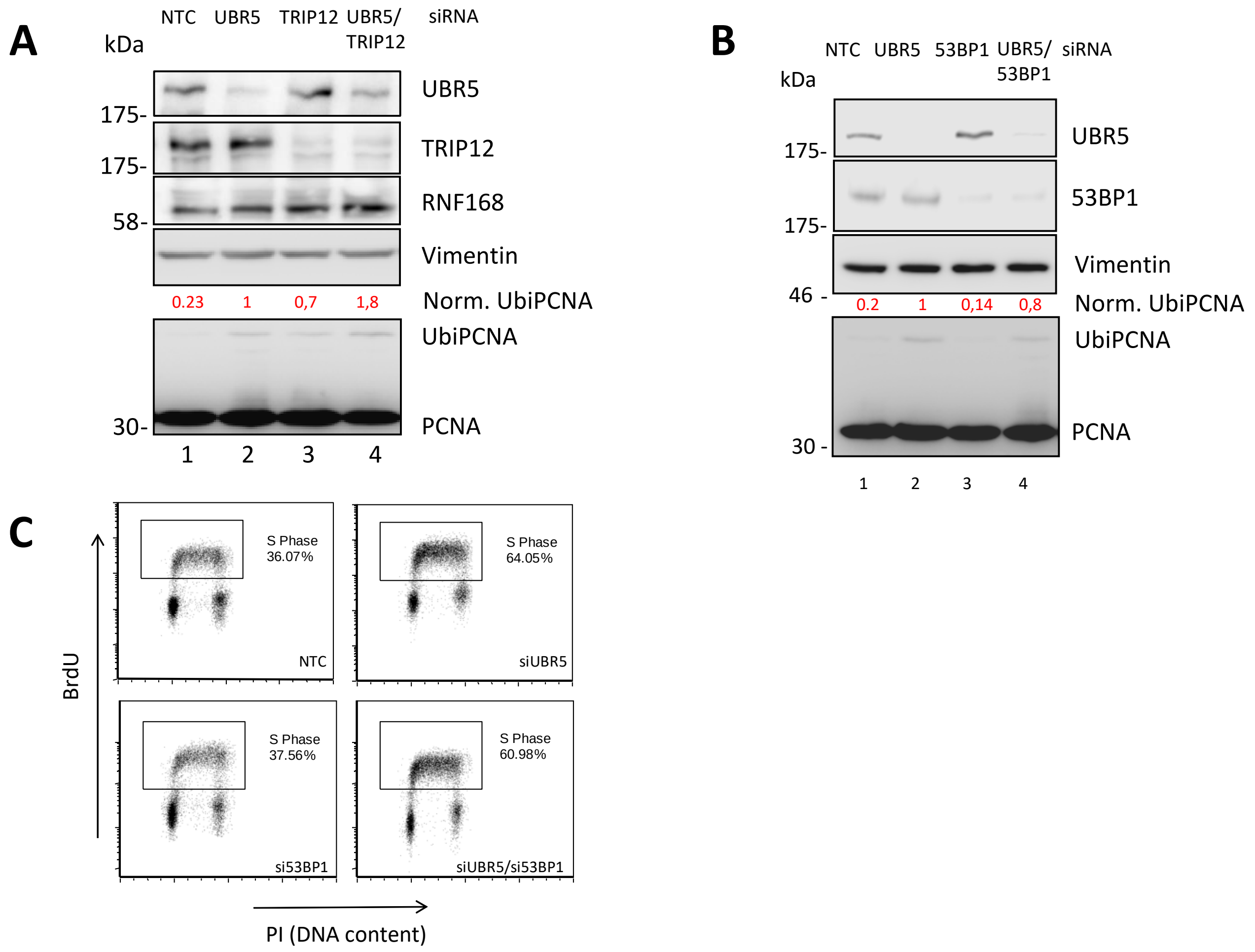

Figure-S4_Cipolla

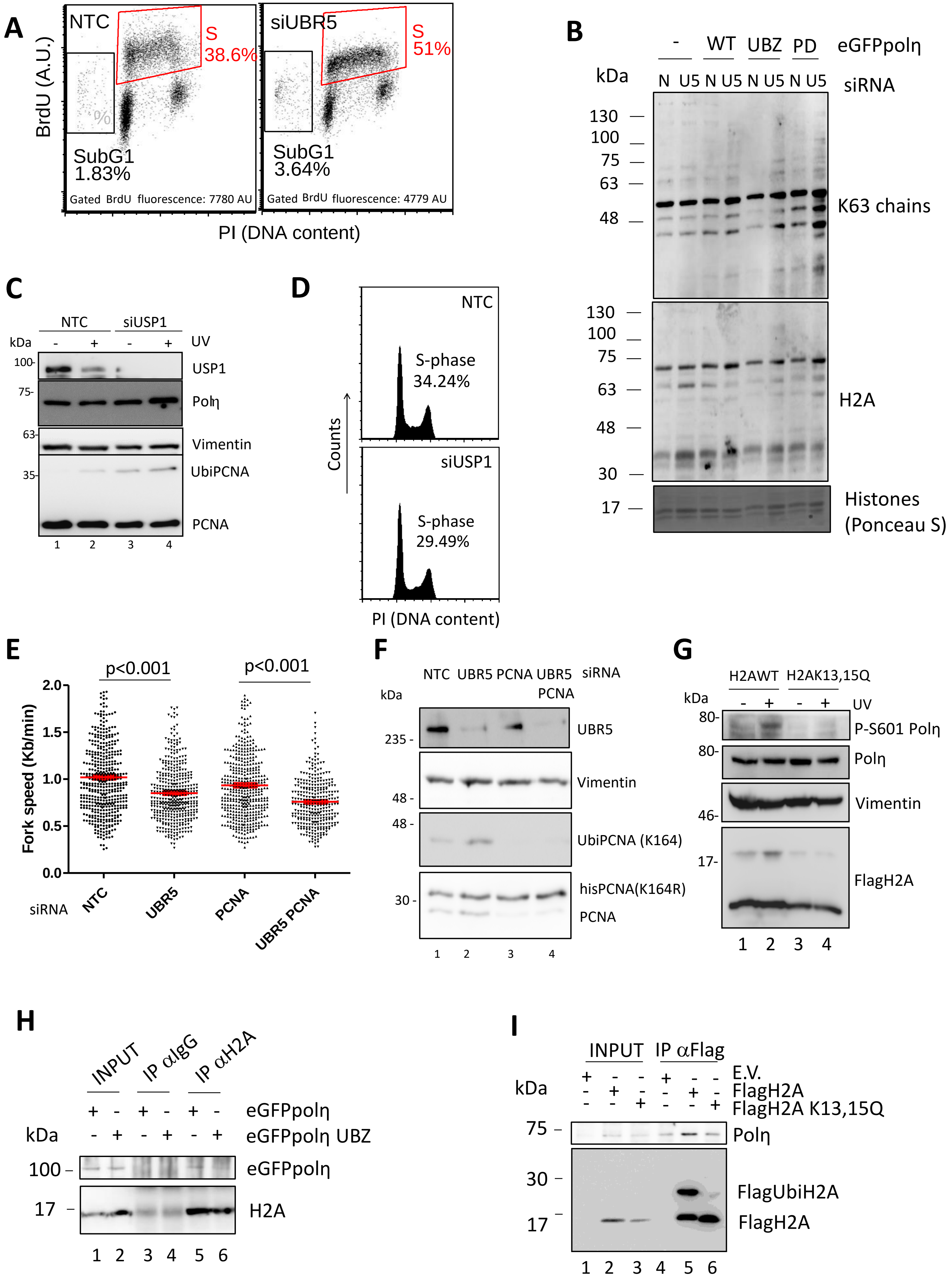

Figure-S5_Cipolla

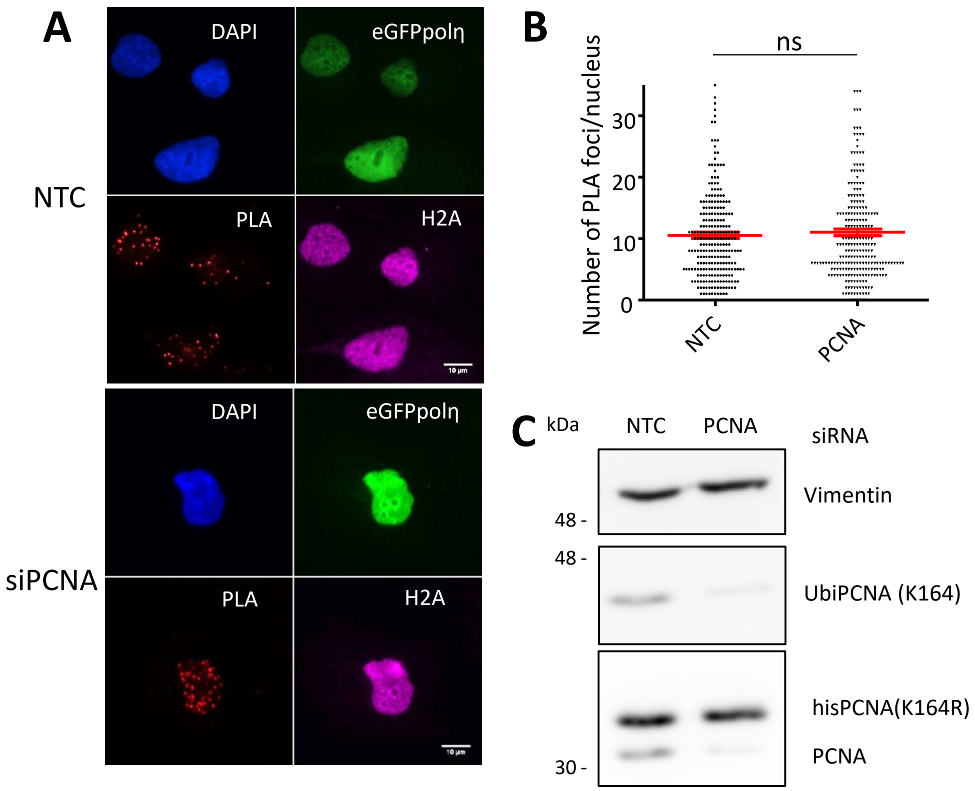

Figure-S6_Cipolla

FigS1. UBR5 silencing effect is specific.

(A) The accumulation of cells in the S-phase of the cell cycle occur after silencing of UBR5 with independent siRNAs. Relative knockdown efficiency, correct for internal loading control, is indicated in the western blot. (B) MRC5 cells silenced with siRNA against UBR5 or NTC for 48h were rescued by transfection with either a plasmid coding for GFP (left) or GFP-UBR5 (right). GFP and GFP-UBR5 cells were gated and analysed by PI staining. Only the expression of GFP-UBR5 resulted in the elimination of the accumulation of the cells in S-phase (red vs black, right panel) as monitored by FACS analysis. (C) Cells silenced for UBR5 or NTC were transfected with GFP alone or GFP-UBR5, either WT or catalytic dead. After 30h GFP positive cells were sorted and cell extracts were analysed by western blot. The numbers on the blot represent the normalized intensity of the UbiPCNA band, corrected for the total amount of PCNA.

FigS2. UBR5 KO obtained by CRISPR/CAS9 recapitulates the observed phenotypes.

(A, B) UBR5 KO cells show an increased S phase population (A) with lower EdU incorporation (B). (C) Distribution plot of the fork speed by DNA fibre assay (n>300 over three experiments) showed a decrease in fork speed in UBR5 KO cells. (D) Western blot analysis of whole cell extracts from WT control and UBR5 KO cells show increase PCNA ubiquitylation when UBR5 was inactivated. (E) Fibre analysis in U2OS cells silenced for UBR5. (F) Western blot analysis of the samples is presented in the E. (G) Analysis of PCNA in S phase sorted cells with DyeCycle Vibrant Orange (Life Technologies) reveals that even in a S-phase homogenous population UBR5 silencing results in increased UbiPCNA. (H) FACS profiles showing the accumulation of cells in S phase, after UBR5 knock down, upon treatment with the ATR inhibitor VE821. (I) As in H but showing the accumulation of UbiPCNA even when ATR is chemically inhibited.

FigS3. Phenotypes caused by alteration of the UbiH2A pathway.

(A) UBR5 silencing results in the accumulation of ubiquitylated histone H2A. Acid extracts were analysed for the presence of either endogenous (left) or transfected FLAG-H2A (right). (B) Ubiquitylated histone H2A increases during the S phase. Acids extract were prepared from double thymidine synchronised XP30RO cells. (C) Modulation of the ubiquitylation of H2A in acid extracts from cells silenced as in Fig4A.(D,E) Concomitant silencing of RNF8 rescues the increase of UbiPCNA (D), the accumulation of the cells in S-phase (E) after UBR5 knockdown. The cells were transfected with the different siRNA for 72h before being harvested and immunoblotted as indicated. Normalized levels of UbiPCNA are indicated in red. In parallel, the S-phase of each knockdown was monitored by FACS analysis.

FigS4. The role of TRIP12 and 53BP1 in during replication.

(A) MRC5 cells were silenced for either UBR5 or TRIP12 or the combination of both for 72h. Protein samples were prepared and immunoblotted as indicated. Normalized levels of UbiPCNA are indicated in red. (B,C) The replication defects are not caused by the accumulation of 53BP1 as monitored by the accumulation of UbiPCNA (B) or FACS analysis (C). Normalized levels of UbiPCNA are indicated in red.

FigS5. **PCNA ubiquitylation *per se* does not cause replication defects.**

(A) A sub-G1 population is present also in MRC5 cells silenced for UBR5, as monitored by FACS. (B) Modulation of the ubiquitylation of H2A in acid extracts from the XP30RO derived cell lines cells silenced as in Fig6E. (C) After 72h of USP1 knock- down (siUSP1) the samples were UV-irradiated or mock treated and immunoblotted as indicated. (D) The cell cycle analysis of unirradiated NTC and siUSP1 cells was monitored by FACS. (E) Fibre analysis, after UBR5 silencing, in a cell line where PCNA cannot be ubiquitylated on K164 (MKR1). (F) Analysis of the levels of UbiPCNA and UBR5 by western blot in the cells used in D. (G) Polη phosphorylation on Serine 601 was monitored in MRC5 cells stably expressing Flag-H2A or Flag-H2AK13,15Q. (H) Endogenous H2A was immunoprecipitated from XP30RO cell lines expressing either eGFPpoη WT or mutated in the UBZ domain. (I) A Flag-H2A vector, either WT of mutated in lysines 13 and 15, was transfected in MRC5 and subjected to αFlag-H2A immunoprecipitation. The immnuprecipitated fraction was probed with a polη antibody to confirm a direct interaction; E.V: Empty Vector.

FigS6. PCNA ubiquitylation does not modulate the interaction between polη and H2A

(A) Proximity between polη and H2A persists in the MKR1 cell line, indicating that the interaction is independent from UbiPCNA. Representative images are shown. (B) Quantitation of the PLA assay (n>200). (C) Analysis of the levels of UbiPCNA in the cells used for the PLA.
